# Practical Impact of the COVID-19 Pandemic on Indoor Air Quality and Thermal Comfort in Kindergartens. A Case Study of Slovenia

**DOI:** 10.3390/ijerph18189712

**Published:** 2021-09-15

**Authors:** Vesna Lovec, Miroslav Premrov, Vesna Žegarac Leskovar

**Affiliations:** Faculty of Civil Engineering, Transportation Engineering and Architecture, University of Maribor, Smetanova ulica 17, 2000 Maribor, Slovenia; vesna.lovec@um.si (V.L.); miroslav.premrov@um.si (M.P.)

**Keywords:** kindergartens, COVID-19, IAQ, thermal comfort

## Abstract

The experimental monitoring of carbon dioxide concentration was carried out in kindergartens in Slovenia, together with indoor air temperature and relative humidity, before and during the COVID-19 pandemic. The aim of the research was to estimate the practical impact of the pandemic on indoor air quality and thermal comfort. The case study sample included buildings with different architectural typology, which are predominantly present in the building stock of Slovenia. The monitoring process lasted for 125 days before and during the COVID-19 pandemic. The results have shown a better indoor air quality in kindergartens during the pandemic, mostly due to ventilation protocols and almost imperceptibly changed indoor air temperature. The COVID-19 pandemic affected air quality in kindergarten classrooms in Slovenia by reducing the average carbon dioxide concentration when children were present in classrooms by 30%.

## 1. Introduction

In December 2019, a new highly infectious virus, i.e., severe acute respiratory syndrome coronavirus (SARS-CoV-2), was discovered in China. By late March 2020, it had become clear that the world was undergoing a global pandemic caused by SARS-CoV-2.

With the COVID-19 outbreak, humanity has been facing challenges, which have had an impact on every aspect of society, health, economy, culture, etc. Changes due to COVID-19 have also had environmental impacts on air quality and pollution [1,2,3], indoor air quality in schools [4,5], energy consumption [6] and on the built environment [7,8,9].

Public buildings are severely affected by the pandemic, especially educational buildings. At the beginning of the new school year 2020–2021, most kindergartens and schools around the world reopened fully, but have since then continued to close and reopen over and over, depending on the epidemiological situation. So far, many facts about the virus and the role of children in its transmission remained controversial and unknown. What has become clear is the fact that society must find a way to live with the virus and to keep educational facilities operating as normally as possible.

Kindergartens are an essential part of modern society by taking care of children, providing a place for social experience and acquiring knowledge. They also provide health and hygiene services, nutrition, mental health and psychosocial support. Apart from their crucial contribution to child development and early education, they are an essential service for employed parents. Therefore, they must operate during the COVID-19 crisis and provide a healthy and safe environment for children. Kindergarten and school closures in March 2020 as a measure for preventing the spread of SARS-CoV-2 had many negative effects on children’s social development, wellbeing, and mental health [10,11].

Kindergartens can be specified as high-density buildings hosting numerous children each day. Therefore, the risk of spreading the virus in such buildings is considered to be high, although after schools reopened, numerous studies have shown that this reopening has not led to an increased risk of COVID-19 outbreaks [12,13]. However, it is extremely important to find the optimal solution for kindergartens to remain open in the COVID-19 era, and to provide a healthy and safe environment for the most vulnerable members of our society. Therefore, numerous protocols and international guidelines were published for school reopening last year [11,12] in order to implement effective measures in order to minimize the risk of transmission. Applied measures inevitably impact the indoor environment, consequently children’s health and wellbeing and their effects on indoor air quality (IAQ) and thermal comfort in kindergartens in Slovenia have been estimated in this research.

### 1.1. Literature Review

In recent years, there have been many studies on indoor environmental quality (IEQ) in educational institutions in different climatic regions [14,15,16,17]. The scientific literature mostly focuses on schools, but few of the available studies target kindergartens [18,19,20]. The topics that have been widely analysed are indoor air quality (IAQ) and thermal comfort. Most studies analyze the impact of indoor environmental conditions on students’ health and wellbeing, as well as on learning processes and academic performance [21,22,23]. Most of the conducted studies report frequent deficiencies in indoor environmental conditions, mainly indoor air quality (IAQ), as a result of inadequate or misused ventilation systems. Most of the research considers IAQ and thermal comfort to be the most important elements of IEQ. Research conducted in Slovenia before the COVID-19 pandemic indicated a high level of CO_2_ which indicates poor IAQ in kindergartens and schools, and inadequate thermal comfort [18,19,20,24]. Despite these findings, neither the institutions nor the public administration have paid special attention to IAQ and thermal comfort in kindergartens and schools.

The COVID-19 pandemic, declared by the World Health Organisation (WHO) on 11 March 2020, has raised renewed interest in the assessment of indoor air quality in high-density buildings, especially school and preschool classrooms, which had to remain open during the pandemic. Numerous studies last year proved that better indoor air quality decreases the risk of coronavirus transmission in indoor spaces. However, no research has examined the practical changes in indoor air quality in kindergartens due to the COVID-19 pandemic.

A currently accessible published study on indoor air quality and thermal comfort in the context of COVID-19 in a primary school in a region with a Mediterranean climate [4] shows that ventilation protocols provide good results in terms of IAQ conditions, but not in relation to IEQ conditions, which are influenced by thermal comfort. The study is based on the analysis of the measured parameters in a new school which used a hybrid model (mechanical and natural ventilation) during the pandemic. There is no research available focusing on IEQ in kindergartens in the context of COVID-19. From this aspect, this research significantly contributes to this currently relevant topic.

Based on the findings of different studies, the transmissibility of SARS-CoV-2 can be influenced by environmental conditions. Indoor environments are the most common places in which SARS-CoV-2 is transmitted and recent research has pinpointed aerosols as one of the transmission routes of SARS-CoV-2. Other studies suggest that SARS-CoV-2 transmission is most commonly via the short-range airborne route [25]. However, it has been confirmed that sharing indoor spaces with one or more infected persons is a major SARS-CoV-2 infection risk [26], and SARS-CoV-2 transmission is exacerbated by poor ventilation [25] and therefore there is a need to improve the hygienic and ventilation conditions of indoor environments to decrease the transmission of airborne infectious diseases [26].

Recent studies also considered the impact of other IEQ parameters on virus transmission. Studies confirmed that in dry indoor places (<40% RH), the chances of airborne transmission of SARS-CoV-2 are higher than in humid places [27], and that cold and dry conditions were potentiating factors for the spread of the virus [28]. Setting the air temperature below 21 °C is not recommended.

Overall, suggesting that enhancing indoor air quality (IAQ) could be extremely effective in reducing aerosol transmission of viruses brings indoor environmental conditions to the forefront, together with ventilation protocols, which have significantly changed during the pandemic.

### 1.2. Main Objectives and Structure

Most of the recently conducted research targeted the nature of the virus and its transmission routes in the indoor environment. Recent research is also very much involved in the impact of IEQ on the spread of the virus. Yet, no available research involves the effect of COVID-19 on indoor conditions in kindergartens. Therefore, the conducted experimental analysis of indoor air quality and thermal comfort is a valuable scientific contribution to the assessment of COVID-19 impact on indoor conditions in kindergartens. This research contributes to a very complex topic that must be constantly updated with the available data that changes daily.

As the world is undergoing global changes due to the COVID-19 crisis, schools and kindergartens have had to adjust to the new reality by implementing protocols and measures to prevent the virus from spreading indoors. New measures target both hygiene and organizational measures, and most importantly measures related to ventilation. At this point the question arises as to how the COVID-19 pandemic and all the implemented measures practically impact IAQ and thermal comfort in kindergartens. Therefore, the objective of the current paper is to analyze IAQ and thermal comfort parameters in kindergartens before and during the COVID-19 pandemic and to provide basic information, which should serve as a starting point for further research.

The content of the current paper is divided into four sections. In Section 1, the background and the objectives of the research are presented in addition to the review of the relevant literature. The review of public health measures and general data on kindergarten buildings in Slovenia is briefly described in Section 2, while in Section 3 the measurement process is presented. The final conclusions are given in Section 4.

## 2. Covid-19 Pandemic and Preschool Education in Kindergartens in Slovenia

### 2.1. Existing Building Stock of Kindergartens in Slovenia

Kindergartens, together with schools, constitute a large share of the public building stock in Slovenia. According to the data of the Ministry of Education, Science and Sport, there is a total of 1177 public and private kindergartens, including all their units, in Slovenia [29]. In addition, there are many children, kindergarten teachers, and other workers, who spend most of their days in kindergartens throughout Slovenia, therefore the issue of IAQ is of extreme importance, with its inevitable impact on children’s health.

Kindergartens in Slovenia are products of various social systems, norms, and construction trends in various historical periods. The average age of kindergartens in Slovenia is over 45 years. The architectural and structural characteristics have followed these construction trends and are very diverse, from massive concrete to light prefabricated structure systems. Buildings also differ in size and in terms of ventilation and heating systems, which is of extreme importance for the existing research. Approximately 88% of Slovenian kindergartens are naturally ventilated [30]. It must also be pointed out that the monitoring of air quality in buildings is virtually non-existent, meaning that teachers must ensure a high-quality indoor environment, which brings the human factor to the forefront. Therefore, special attention must be paid to the ventilation of classrooms throughout the day, especially in the ongoing pandemic circumstances.

The outbreak of COVID-19 has significantly affected preschool education, work processes have had to be adapted, and buildings themselves have undergone certain adaptations to carry out organizational measures to contain the spread of the virus. The most significant changes which can potentially impact occupants’ health by improving IAQ is a changed ventilation protocol, which is presented in more detail below.

### 2.2. Public Health Measures for Kindergartens Associated with COVID-19

After a two-month total lockdown, kindergartens gradually reopened in May 2020. In September 2020, kindergartens fully reopened in most countries worldwide with consideration given to public health measures for kindergartens associated with COVID-19.

International guidelines and recent research regarding school reopening have determined the need to implement effective measures to minimize the risk of transmission, including face masks, interpersonal distance of two meters, frequent hygiene measures, and improved ventilation [10,11,12,31,32]. Guidelines published for schools are applicable also to kindergartens, although obstacles that occur in practice should be mentioned: distancing is difficult to maintain, kindergarten classrooms cannot be ventilated during breaks like school classrooms, young children do not understand why measures have to be implemented, and it is more difficult for them to cooperate, etc. According to the recent research, natural, mechanical, and hybrid ventilation systems can be used effectively and safely in schools, removing contaminated indoor air and supplying fresh outdoor air [12].

Based on ample research, the WHO announced that there is mounting evidence that airborne transmission of COVID-19 is possible indoors, especially in poorly ventilated spaces. Therefore, ventilation protocols have been recognized as crucial to reduce transmission in indoor spaces, and they are treated as an important measure for preventing the virus from spreading indoors. They have been implemented immediately in educational institutions worldwide as public health measures to prevent virus transmission.

Public health measures and recommendations for kindergartens and schools to prevent the spread of the virus are adjusted locally by government institutions. Generally, they include two groups of basic measures: (1). instructions regarding the sanitary and hygiene regimes in buildings (room hygiene, hand hygiene, cough etiquette, disinfection, ventilation, mask-wearing, etc.), and (2). organizational measures (instructions on maintaining physical distance, aiming to reduce contact between the occupants). Most important for this research are the instructions which impact indoor air quality.

In Slovenia, the National Institute of Public Health of the Republic of Slovenia issued ”Hygiene-Related Recommendations for Kindergartens to Prevent The Spread of Sars-Cov-2”, which address all segments of kindergarten functioning during the COVID-19 pandemic [33]. The section on the cleaning and ventilation of buildings in the issued recommendations refer to ”Instructions for Building Ventilation Outside of Healthcare Centres During the Spread of COVID-19”, updated on 23 April 2021. These instructions provide general guidance on ventilation, which affects air quality and also thermal comfort.

The basic guidance or recommendations related to ventilation protocols published by the National Institute of Public Health, Lublijana, Slovenia (NIJZ), which should be observed by all kindergartens in Slovenia, and affect air quality in kindergartens, are shown in Table 1. Besides, basic recommendations related to thermal comfort are also presented.

All the provided data were issued as recommendations in Slovenia to be observed by kindergartens. With the assumption that all Slovenian kindergartens fallowed the recommendations to prevent the spread of SARS-CoV-2, this research experimentally analyses changes in the indoor environment parameters related to IAQ and thermal comfort, due to the pandemic. The question of its impact on occupants health and wellbeing still remains open.

National Slovenian legislation remains unchanged, in terms of IEQ, before and during the pandemic. Legislation governing air quality and thermal comfort in buildings prescribed CO_2_ concentration at ≤1667 ppm, and air temperature between 19 °C and 24 °C [34] and a minimum of 20 °C in kindergarten classrooms [35]. The prescribed humidity is between 30% and 70% [34].

## 3. Measurements In Situ—Research into the Thermal Comfort and IAQ in Kindergartens before and during the COVID-19 Pandemic

### 3.1. Methodology

The aim of the research is to estimate the effects of the COVID-19 pandemic on the indoor air quality of kindergartens in Slovenia. For this reason, the methodology of the study follows three basics steps: 1. collecting data on the IAQ and thermal comfort parameters by in-situ measurements before and during the COVID-19 pandemic; 2. comparing and analyzing collected data in relation to building characteristics, number of occupants, ventilation protocols, etc., before and during the pandemic; and 3. discussion and conclusions.

**Figure 1:**
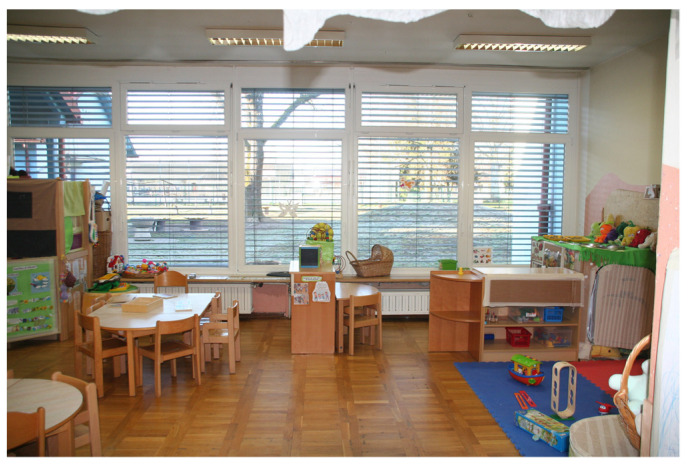
Kindergarten classroom, 2A measured interval.

The research methodology covered experimental analyses of individual parameters of indoor air quality and thermal comfort in kindergartens in Slovenia in the winter before and during the COVID-19 pandemic. Carbon dioxide concentration CO_2_ (ppm) together with indoor air temperatures T_ai_ (C°) and indoor relative humidity RH_ai_ (%) were monitored with continued in situ measurements. The data on outdoor air temperatures and humidity are summarized from the archives of the Slovenian Environment Agency. As part of the measurements, records of the number of children present in classrooms and ventilation intervals were kept every day in addition to air quality and thermal comfort parameter monitoring. The measurements were carried out in eight kindergartens in the winter before and during the COVID-19 pandemic in Slovenia for 125 days, i.e., 69 days in the 2019–2020 heating season before the COVID-19 pandemic, and 56 days in the 2020–2021 heating season during the COVID-19 pandemic (Figure 1). Parameters measured in the occupation interval were analysed, and data collection corresponding to holidays and non-school days have been removed in order to ensure minimal distortion of the results.

As the measurements were carried out, activities in the classrooms were uninterrupted, and the teachers were asked not to change their daily routine, which was slightly different before and during the COVID-19 pandemic, particularly in the way the buildings were ventilated. Before the COVID-19 pandemic, Slovenian kindergartens had established protocols for natural ventilation, while after the outbreak, they observed the NIJZ instructions based on the WHO recommendations.

**Figure 2:**
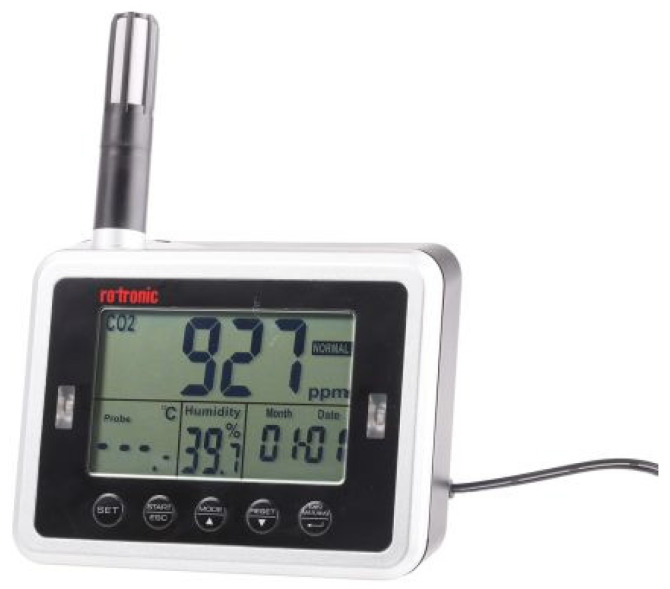
Benchtop display data logger rotronic CL11 unit.

Data loggers were placed so that the sensors were not directly affected by heat sources (radiators) or sunlight, and were outside of the children’s breathing zone (which is at a height of between 0.5 and 0.7 m), i.e., at a height of approximately h = 1.5 m. The sensors were placed away from windows in the middle of the classroom and approximately 0.6 m from the wall. The data logger rotronic CL 11 (Rotronic Instruments, Crawley, UK) (Figure 2) was used to measure T_ai_, RH_ai_ and CO_2_ [36], with an infrared sensor (NDIR) with automatic calibration for CO_2_ measurements, and a humidity sensor ROTRONIC HYGROMER^®^ IN-1 (Rotronic Instruments, Crawley, UK) and NTC (Rotronic Instruments, Crawley, UK) thermistor sensor for humidity measurements The data from the data logger is shown in Table 2.

### 3.2. Case Study Buildings

The analysed sample included eight purpose-built kindergartens (Table 3). Like all European countries, Slovenia has a diverse kindergarten building stock, which is why a sample of buildings with diverse architectural, structural, and energy-related characteristics was selected for this research. The aim is to include, with a carefully selected sample, as many different types of building as possible, providing general conclusions on the effects of the COVID-19 pandemic on air quality and thermal comfort in kindergartens via the collected data.

The sample of buildings is included evenly in both measuring periods, before (Measuring period A) and during (Measuring period B) the COVID-19 pandemic. The measurements were carried out in four architectural types of building (marks 1 to 4 of measuring intervals in the table), in two measuring periods, in twelve classrooms, for 125 days in two heating seasons. In each measuring period, one building with the same characteristics (concrete/wooden, natural/forced ventilation, ground floor/several floors) was the subject of the measurements. The buildings were built in various periods between 1950 and 2010 with different structural systems, different sizes, different classroom orientation, and with different heating and ventilation systems. The sample ensured approximately equal conditions in all parameters of the selected sample in both measuring periods, before and during the COVID-19 pandemic.

## 4. Results

The analysis of the results included a comparison of data measured in the measuring periods before (Measuring period A) and during (Measuring period B) the COVID-19 pandemic in kindergartens in Slovenia. The monitoring included four different architectural types of kindergarten marked 1 to 4, which are the most commonly represented in the kindergarten stock (measurements 1 to 4). The table shows the parameters before and during the COVID-19 pandemic measures when the children were present in the classrooms: relative humidity RH_ai_ (%), air temperature T_ai_ (°C), and carbon dioxide concentration CO_2_ (ppm) (Table 4). The represented values correspond to average values and maximum and minimum measured parameters during the children’s presence in the kindergarten. Standard deviation of all measured parameters for air temperature, humidity and carbon dioxide for all measured parameters before and during the COVID-19 is presented.

For a comprehensive analysis of thermal comfort parameters, outdoor air parameters are also recorded in this research, i.e., outdoor air temperature T (°C), and humidity H (%), which are shown in Table 5.

The two most important variables which had impact on the IAQ as well as on thermal comfort are ventilation rates and number of occupants. Both varied between two measuring seasons due to the CVID-19 pandemic, and therefore were recorded. Detailed information on heating regimes was not available.

Ventilation is the crucial element affecting air quality. For this reason, the sample of the actual ventilation in practice was monitored during the implementation of the measurements. In the 2019–2020 heating season before the COVID-19 pandemic, the classrooms were ventilated during the day in line with established practice, determined by each kindergarten individually. The dynamics and intensity of ventilation were adjusted by teachers. In the 2020-2021 heating season, ventilation was slightly more intensive according to the previously presented instructions for building ventilation outside of healthcare centres during the spread of COVID-19. In the selected sample of buildings, only buildings in the measuring process marked with 4 have mechanical ventilation, which was the only type of ventilation before the pandemic, while during the pandemic, the buildings were ventilated with a combination of mechanical and natural ventilation. The sample of classroom ventilation is presented on the basis of the actual sample recorded in situ (Table 6). The children arrive at the classroom between 6 a.m. and 7 a.m., depart between 3 p.m. and 3.30 p.m., and ventilation intervals are between 10 and 30 min. In the measuring period during the COVID-19 pandemic, ventilation intervals were notably more frequent and lengthier.

The number of children present is the second parameter that can strongly affect air quality in a room. This is why the number of children was recorded at the time of the measurements in the classrooms. In terms of IAQ and thermal comfort, an important parameter related to the number of occupants is also classroom floor area (m^2^) presented in Table 4. The maximum number of children in a classroom is in line with Slovenian standards. This research shows the share of children present in relation to the prescribed norm (which presents a full capacity of classroom occupancy) for individual measurements 1–4 in each measuring period (Table 7), together with age of children, classroom floor area and number of children enrolled.

**Figure 3:**
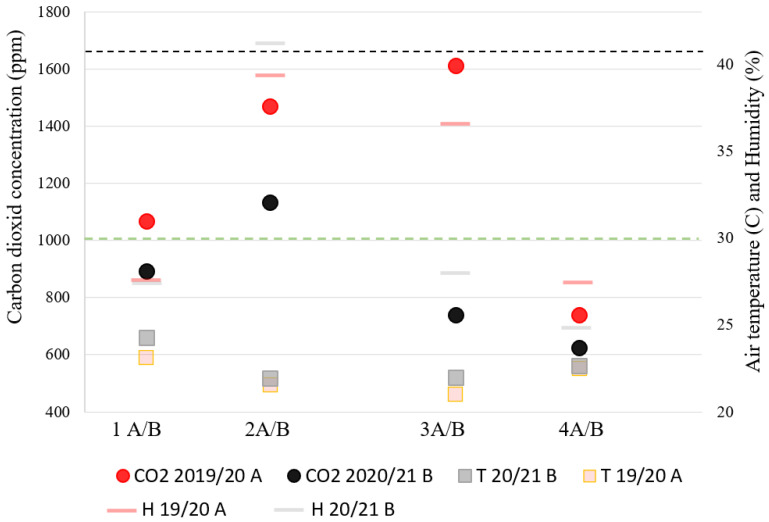
Measured parameters before and during the COVID-19 pandemic (CO_2_—carbon dioxide average concentration, T—air temperature, H—relative humidity; 2019–2020 A—first measuring period, 2020–2021—second measuring period).

The COVID-19 era brought numerous changes to Slovenian kindergartens together with ventilation protocols and decreased number of occupants. All these changes inevitably impact indoor air quality and thermal comfort, which is the focus point of this research. The most dramatic difference in CO_2_ concentration before and during the pandemic is within the measures 3 A/B, naturally ventilated, prefabricated kindergartens from the early 1970s (Figure 3). The smallest fluctuations in the measured parameters are, as expected, in mechanically ventilated buildings. A clear relation between improvement in IAQ and changes in thermal comfort parameters cannot be established.

Measured results clearly indicate daily average measured parameters of CO_2_ concentration in range with Slovenian legislation (≤1660 m), both before and during the pandemics. CO_2_ concentration before the pandemic was mostly not within the range recommended by international documents and standards (≤1000 ppm). Indoor air temperature reached the recommended value of >20 °C. Air humidity was below the recommended values. However, update of the legislation related to indoor air and thermal comfort should be considered in future.

## 5. Discussion

The analysis of the results encompasses a comparison of the measured data in two measuring periods, i.e., before and during COVID-19 pandemic in Slovenia. Based on the analysed sample of buildings over a total of 125 days and on the assumption that all kindergartens followed ventilation protocols, hygiene, sanitary and organizational measures, conclusions are as follows:

- The COVID-19 pandemic affected air quality in kindergartens in Slovenia by reducing the average carbon monoxide concentration when children were present in classrooms by 30%, based on the average of the measured parameters in the buildings analyzed in this research This is a consequence of more intensive ventilation defined by the protocols related to COVID-19 prevention and decreased number of children.
- Due to the actual illness of children and family members, or parents’ fear of contracting SARS-CoV-2, there were fewer children present in the classrooms in kindergartens in Slovenia during the COVID-19 pandemic than before the pandemic. Analyses have shown that the average occupancy of the classrooms in the heating season before the COVID-19 pandemic was 83%, while the capacities were only 72.5% full during the pandemic. The fact is that 10% less children in the classrooms partially affects indoor air quality in addition to a better ventilation protocol, which is the primary factor underlying better air quality. In the measurements for no. 3, the greatest difference in the occupation of the classrooms at approximately 20% is also the greatest difference in the average measured carbon dioxide concentration before and during the COVID-19 pandemic.
- When comparing the measures’ parameters, the smallest differences before and during the COVID-19 pandemic could be noticed in buildings with forced/mechanical ventilation, in which air quality had been within the statutory limits and in accordance with the recommendations of experts even in the season before the pandemic. During the pandemic, ventilation in these buildings was a combination of natural and mechanical ventilation with fresh air supply, which further improved air quality, and did not affect thermal comfort, although heat losses were expected to be greater, which will be the subject of further research.
- In various buildings, different parameters of air quality were measured, influenced mostly by the ventilation protocol and number of occupants. All buildings followed the same ventilation protocols, but the results directly depend on the implementation procedure and the human factor, and on the awareness of users of the significance of ventilation, which is insufficiently discussed in the scientific literature. Users of these spaces are extremely important in terms of the implementation of measures, and should be educated in terms of ventilation procedures and their impact on indoor air quality.
- Analyses have shown that higher intensity of classroom ventilation did not result in poorer thermal comfort in the classrooms, since the average temperature dropped by a mere 0.12 °C in the measuring periods before and during the COVID-19 pandemic. The difference in the average outdoor temperature between the two measuring periods was 0.94 °C. The heating regime was unchanged. Potential major heat losses resulting from more intensive natural ventilation will be the subject of further research. The difference in the average measured air humidity is approximately 2%. Implemented measures inevitably also impact other indoor environmental parameters, which will also be the issue of further research.
- In both measuring periods, the average CO_2_ concentration was within the limits stipulated by Slovenian legislation, i.e., ≤1667 ppm, but exceeded the recommendations (≤1000 ppm), apart from mechanically ventilated buildings, which had satisfying air quality both before and during the pandemic. The measured daily average air temperatures are slightly higher, but within the statutory limits. The air in the classrooms was dry, which is problematic from the aspect of the spread of the virus and exceeds the recommendation of ≥40%.

Last year, there were numerous changes in the operational process of kindergartens. That with the most dramatic consequences on the IAQ and thermal comfort is the ventilation protocol, which especially impacts naturally ventilated kindergartens. Since naturally ventilated kindergartens made up the majority of the kindergarten building stock, not only in Slovenia but in the Balkan countries (Serbia, Croatia, Montenegro, North Macedonia) and most Eastern Europe, this study is of great importance. Finally, the results and conclusions of this study should be potentially used for upgrading and developing further ventilation strategies and protocols. Although this study has limitations, monitoring process was challenging and faced many obstacles during the pandemic. Public health measures and recommendations for kindergartens required restricted movement in classrooms and most of the evidence depended on teachers.

## 6. Conclusions

This study has analysed the effects of the COVID-19 pandemic on IAQ and thermal comfort conditions in kindergartens in Slovenia in the winter period. Indoor environmental variables, i.e., CO_2_ concentration levels together with temperature and humidity, were monitored and analysed before and during the pandemic.

During these years, the accomplishment of adequate thermal comfort in kindergartens and schools together with energy consumption in relation to thermal comfort was prioritized over achieving a satisfying IAQ. COVID-19 has evidently brought the importance of the IAQ to the forefront. Special attention to IAQ led to significant changes in ventilation protocols, guidelines, and IAQ requirements for public buildings last year due to the concern over the transmission of SARS-CoV-2. During the pandemic, it became mandatory to supply fresh air using manual airing, irrespective of outdoor conditions and heat loses.

Based on the analysed sample of kindergartens in Slovenia, ventilation protocols related to the prevention of COVID-19 transmission provide good results in terms of IAQ. A comparison of the results measured in Slovenian kindergartens shows a 30% improvement in the average daily concentration of CO_2,_ while thermal comfort was not at risk during the COVID-19 pandemic. Heat losses and additional expenses for heating are expected and could be the subject of further research.

The research has shown improved indoor air quality in kindergartens due to protocols related to the COVID-19 pandemic and a decreased number of occupants. During their stay in kindergartens in the winter season during the pandemic, children were exposed to better indoor air quality in the classrooms. which will inevitably impact their health and wellbeing. Time will tell whether the improved IAQ will become standard in indoor spaces, and it will be years before the far-reaching consequences of the impact on children’s health are revealed. The paradoxical question remains: can we consider that COVID-19 brought some improvement in health and wellbeing conditions in our kindergartens?

## Figures and Tables

**Table 1:** Recommendations for kindergartens to contain the spread of SARS-CoV-2.

Protocols/Recommendations Regarding Air Quality and Thermal Comfort	
Natural vntilated buildings	- Regular opening of windows wider than usual, even if this causes certain thermal discomfort. - Opening of windows to support correct ventilation before and after a space is occupied by several people Windows must be open for approximately 15 min before people can enter a closed space in non-residential buildings. - Recommended minimum air change of 6–12 changes per hour (5–6 for schools).
Mechanically ventilated buildings	- Constant ventilation through fresh air supply with the highest possible air flow, which does not circulate within the room.
**Recommendations Regarding Thermal Comfort**	
All buildings	- Setting the temperature below 21 °C and humidity below 40% is not recommended, as these are optimal conditions for the survival of SARS-CoV-2.

**Table 2:** Data from the data logger and the measurement process.

Parameter	Units	Accuracy	Limit Range	Measured Interval
CO_2_ concentration	ppm	±30 ppm (±5%)	0–5000	15 min
Air temperature	°C	±0.3	−2—60	15 min
Relative humidity	%	±2	0–100	15 min

**Table 3:** Case Study Buildings (B—basement, G—ground floor, B + G + 2—basement + ground floor + 2 stories).

Sample of Buildings	2019–2020	2020–2021
	A. Measuring period before COVID-19	B. Measuring period during COVID-19
–B + G + 2 –period of construction: 1970–1980 –solid structural system –natural ventilation –classroom orientation: south/east	1. A Measured interval– January 2020 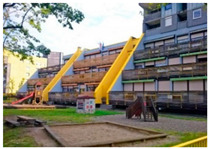	1. B Measured interval— February 2021 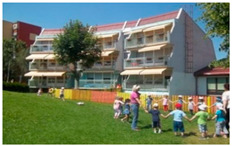
–G – period of construction: 1950–1960 –solid structural system –natural ventilation –classroom orientation: south-east/south	2. A Measured interval— February 2020 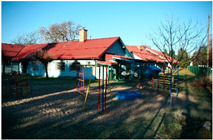	2. B Measured interval— February 2021 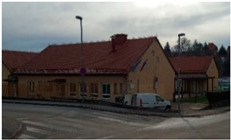
–G –period of construction: 1960–1970 –prefabricated structural system –natural ventilation –classroom orientation: south-east/south	3. A Measured interval— January 2020 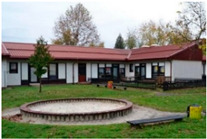	3. B Measured interval— February 2021 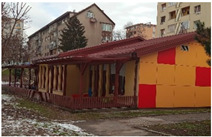
–G to G + 2 –period of construction: 1980–2010 –solid structural system –mechanical ventilation - classroom orientation: south-east/east	4. A Measured interval— February 2020 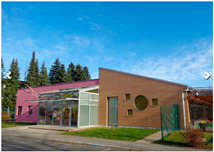	4. B Measured interval— February 2020 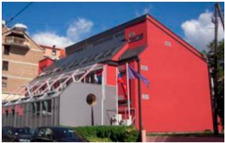

**Table 4:** Measured parameters: air temperature Tai, relative humidity RHai, and carbon dioxide concentration CO2 (MIN—minimum measured value; MAX—maximum measured value; AVG—average measured value during the children’s presence in the kindergarten) in the winter period before and during the COVID-19 pandemic.

No. of Measurements		T (°C)			CO_2_ (ppm)			H (%)		
		MIN	MAX	AVG	MIN	MAX	AVG	MIN	MAX	AVG
Before COVID-19 pandemic measurements	1A	18.22	24.83	23.11	410	2452	1067.38	16.9	39.3	28.63
	2A	18.97	23.44	21.57	441.50	2936.5	1468.44	24.45	49.35	39.37
	3A	18.72	23.17	21.13	372	2673	1612.02	26.9	44.4	36.82
	4A	19.91	23.66	22.50	373	1581.5	739.69	15.6	40.95	28.46
standard deviation of all measuring parameters		1.65 T (°C)			535.30 CO_2_ (ppm)			6.62 H (%)		
2019/2020		18.22	24.83	22.0775	372	2936.5	1221.88	15.6	49.35	33.32
During the COVID-19 pandemic measurements	1B	21.56	27.22	24.25	405	2213	891.81	18.5	43.1	28.44
	2B	19.165	23.305	21.95	427	1843.5	1132.84	25.85	55.35	42.99
	3B	16.89	26.72	21.96	388	1947	739.018	17.9	48.6	28.06
	4B	17.47	25.47	22.65	403.5	1151.5	624.8	16.4	35.6	25.01
standard deviation of all measuring parameters		2.10 T (°C)			377.30 CO_2_ (ppm)			11.62 (%)		
2020–2021		16.89	27.22	21.955	388	2213	847.12	16.4	55.35	31.125

**Table 5:** Outdoor air parameters: air temperature T_ai_, relative humidity RH_ai_ (MIN—minimum measured value, MAX—maximum measured value, AVG—average measured value during the children’s presence in the kindergarten).

Measuring Period	AVG. T (°C)	Min. T (°C)	Max. T (°C)	AVG. H (%)
2019–2020	3.84	−8	21	73.36
2020–2021	4.77	−9	22	69.52

**Table 6:** Average sample of ventilation in kindergartens in the 2019/20 measuring period (buildings 1–3, building no.4 has forced ventilation) before the pandemic and in 2020–2021 (buildings 1–4) during the COVID-19 pandemic.

Daily Timeline—Arrival of Children to the Classroom between 6:00 a.m. and 7:00 a.m., Departure between 3:00 p.m. and 3.30 p.m.																			
	7:00 a.m.				9:00 a.m.				11:00 a.m.				1:00 p.m.				3:00 p.m.		
2019–2020																			
2020–2021																			

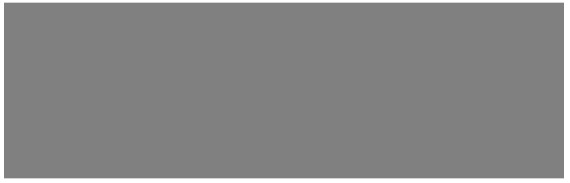
 Ventilation intervals, based on evidence in situ.

**Table 7:** Occupation of classrooms, share of children present in relation to the prescribed norm, maximum number of children enrolled and classroom floor area.

Occupation of Classrooms	2019–2020	2020–2021
	A. Measuring period before COVID-19	B. Measuring period during COVID-19
	1. A Measured interval– January 2020	1. B Measured interval– February 2021
No of children enrolled + teachers	14 + 2	11 + 2
Maximum no of children enrolled (prescribed norm)	14	14
Age of children	2–3 years	2–3 years
Average occupation of classrooms, the share of present children in relation to the prescribed norm	75%	81%
Classroom floor area (m^2^)	51	49.8
	2. A Measured interval– February 2020	2. B Measured interval– February 2021
No of children enrolled + teachers	14 + 2	11 + 2
Maximum no of children enrolled (prescribed norm)	21	21
Age of children	4–6 years	4–6 years
Average occupation of classrooms, the share of present children in relation to the prescribed norm	88%	78%
Classroom floor area (m^2^)	45.5	48
	3. A Measured interval– January 2020	3. B Measured interval– February 2021
No of children enrolled + teachers	24 + 2	21 + 2
Maximum no of children enrolled (prescribed norm)	24	24
Age of children	3 years	3 years
Average occupation of classrooms, the share of present children in relation to the prescribed norm	83%	60%
Classroom floor area (m^2^)	43	40
	4. A Measured interval– February 2020	4. B Measured interval– February 2021
No of children enrolled + teachers	24 + 2	23 + 2
Maximum no of children enrolled (prescribed norm)	24	24
Age of children	5–6 years	5–6 years
Average occupation of classrooms, the share of present children in relation to the prescribed norm	86%	71%
Classroom floor area (m^2^)	59.2	56.1
